# Prioritizing the risk of cervical cancer: findings from the SCCUT Multicentre Initiative

**DOI:** 10.25122/jml-2025-0100

**Published:** 2025-06

**Authors:** Gabriel Marian Saveliev, Valentin Nicolae Varlas, Madalina Piron-Dumitrascu, Nicolae Suciu

**Affiliations:** 1 National Institute for Mother and Child Health Alessandrescu-Rusescu, Bucharest, Romania; 2 Carol Davila University of Medicine and Pharmacy, Bucharest, Romania; 3 Filantropia Clinical Hospital of Obstetrics and Gynecology, Bucharest, Romania

**Keywords:** Cervical cancer, HPV high-risk, HPVhr testing, Pap test, cervical screening, SCCUT, women's health, Romania, cytology, primary prevention

## Abstract

Cervical cancer (CC) remains a significant global health burden, ranking eighth in incidence and ninth in cancer-related mortality among women worldwide. Persistent infection with high-risk human papillomavirus (HPVhr) is the primary etiological factor in CC development. The SCCUT program in Romania was designed to enhance access to primary screening and assess HPVhr prevalence among women aged 25–65 years in the South-Muntenia region. Between August 2022 and November 2023, a total of 36,813 women were enrolled in the SCCUT program after providing informed consent. Based on age, participants underwent either a Pap test and/or HPVhr testing. Clinical and demographic data—including age, hormonal status, intrauterine device (IUD) use, presence of leucorrhea, and history of cervical interventions—were collected via structured questionnaires. HPVhr testing was performed using the AmpFire^®^ HPV Screening system and processed in the lead partner laboratory. Of the total cohort, 12.5% (*n* = 4,588) tested positive for HPVhr. HPVhr positivity was significantly associated with younger age (mean 45.12 ± 9.21 years vs. 46.28 ± 8.56 years; *P* < 0.001) and cyclic hormonal status (*P* < 0.001). No statistically significant associations were found between HPVhr status and IUD use, presence of leucorrhea, or prior cervical interventions (biopsies or cauterizations). The SCCUT program confirms the critical role of HPVhr in cervical carcinogenesis and supports its use as a first-line screening tool. These findings highlight the importance of implementing widespread primary HPVhr testing, particularly in underserved populations. Increasing public awareness, improving HPV vaccination rates, and expanding access to early detection programs remain essential for effective cervical cancer prevention in Romania and globally.

## INTRODUCTION

Cervical cancer (CC) remains a significant challenge to women’s health globally. It ranks eighth among all human cancers, with 662,301 new cases annually, and ninth in global cancer-related mortality, accounting for 348,874 deaths [1]. The epidemiology of CC, currently marked by significant reductions in incidence and mortality, reflects the benefits of newly implemented cervical screening programs, now functional in many countries. However, national CC prevention programs may lead to marked fluctuations in disease eradication, depending on the timing of their implementation. A deeper understanding of CC etiology is essential for shaping the global initiative aimed at eliminating the disease [2].

**Figure 1:**
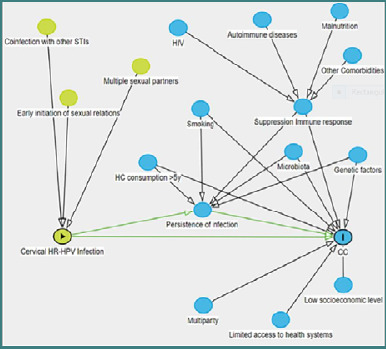
Risk factors for HPVhr infection and persistence [5]

Over the past decades, extensive research has established that persistent infection with high-risk human papillomavirus (HPVhr) is the principal cause of CC, being implicated in 99.7% of cases. As a sexually transmitted infection, HPV has confirmed CC as the most prevalent cancer with a viral etiology [3,4]. A Several cofactors are known to potentiate HPVhr persistence and progression to malignancy, including early onset of sexual activity, multiple sexual partners, multiparity, co-infections with other sexually transmitted pathogens (e.g., *Chlamydia trachomatis*, HSV-2, HIV), genetic predispositions, disruption of the cervical-vaginal microbiome, nutritional deficiencies, immunosuppressive conditions, smoking, hormonal contraceptive use, and poor access to health education and medical services (Figure 1) [5,6].

Currently, HPV—characterized by cutaneous and mucosal tropism, with a preferential affinity for the cervix—has been classified through genomic analysis into over 210 genotypes (International Human Papillomavirus Reference Center), which are further categorized as high-risk (hr) or low-risk (lr) based on oncogenic potential. Of these, more than 40 genotypes (hr ± lr) are capable of infecting mucosal surfaces and genital integuments, frequently leading to benign, borderline, or malignant cervical lesions [7]. The genotypes HPV16 and HPV18 (highly oncogenic), along with HPV6/11/16/18/31/33/45/52/58, are implicated—either individually or in combination—in over 90% of cervical cancer cases, supporting their etiologic role in CC pathogenesis [8]. The Pap smear (PAP), introduced in 1941, represented the initial milestone in early cervical cancer detection. However, the discovery in the 1980s that HPV is the primary etiological agent of CC led to the development and implementation of HPV vaccination and testing as first-line tools in cervical cancer screening. Today, HPVhr testing and liquid-based cytology are widely recommended and globally adopted. The confirmation of HPVhr as the primary cause of CC catalyzed the formulation and launch of a comprehensive global strategy for its prevention, control, and eventual elimination—an initiative formally endorsed by the World Health Organization on November 17, 2020 [5,9,10].

In Romania, the SCCUT program (Screening pentru Cancerul de Col Uterin și Tratament precoce – Cervical cancer screening and early treatment) (August 2020–November 2023) introduced HPVhr testing as the first-line method in primary cervical screening. The program aimed to establish an accessible, organized healthcare framework focused on the prevention, screening, early diagnosis, and treatment of cervical cancer among women in the South-Muntenia region, encompassing seven counties [5,11]. The objective of this study was to highlight the presence of HPVhr as a primary risk factor in the initiation, development and negative evolution of CC, an aspect detailed in the SCCUT tests.

## MATERIAL AND METHODS

This cross-sectional study was conducted within the framework of the SCCUT program (August 2022 – November 2023), which targeted women from the South-Muntenia region of Romania. A total of 36,813 women aged 25–65 years were enrolled following informed consent, which included agreement for voluntary participation, confidentiality, and testing for Papanicolaou (Pap) cytology and/or high-risk human papillomavirus (HPVhr). All participants received structured information regarding HPV, cervical cancer (CC) risk, and the importance of cervical screening, They also completed a standardized questionnaire that included demographic, clinical, and gynecologic history variables: age, hormonal status (cyclic vs. menopausal), presence of intrauterine devices (IUDs), presence of leucorrhea, and prior cervical procedures (biopsies, cauterizations). According to SCCUT screening protocols and current clinical guidelines, women aged 25–29 years underwent initial Pap cytology, with reflex HPVhr testing where indicated. Women aged 30–65 years underwent primary HPVhr testing, with reflex cytology based on the HPVhr result [8]. Cervical samples were collected and transported under standardized SCCUT procedures to the central laboratory at the “Alessandrescu-Rusescu” National Institute for Mother and Child Health (INSMC), Bucharest. Pap cytology was performed using the ThinPrep^®^ and ThinPrep Imaging System. HPVhr testing was conducted using the AmpFire^®^ HPV Screening 16/18/HR assay (Atila BioSystems 4.1) on the PowerGene 9600 Plus Real-Time PCR platform [12,13].

### Statistical analysis

Descriptive and comparative statistical analyses were performed to evaluate associations between high-risk human papillomavirus (HPVhr) status and various demographic and clinical characteristics. Continuous variables, such as age, were expressed as mean ± standard deviation (SD) and compared between HPVhr-positive and HPVhr-negative groups using the independent samples Student's *t*-test. Categorical variables, including age categories, hormonal status, intrauterine device (IUD) use, presence of leucorrhea, and pre-screening cervical history (cauterizations and biopsies), were reported as counts and percentages, and compared using Fisher’s exact test. A *P* value of < 0.05 was considered statistically significant. Statistical analyses were conducted using standard software tools appropriate for epidemiological cohort analysis.

## RESULTS

Among the 36 813 women aged 25–65 years enrolled in the SCCUT study, 4 588 (12.5 %) tested positive for high-risk human papillomavirus (HPVhr) and 32 225 (87.5 %) tested negative. The mean age of the cohort was 46.14 ± 8.65 years; HPVhr-positive women were significantly younger than HPVhr-negative women (45.12 ± 9.21 vs 46.28 ± 8.56 years; *P* < 0.001, Student *t*-test). HPVhr positivity was more common in the 25–29 year age band than in the 30–65 year band (3.1 % vs 1.0 %; *P* < 0.001, Fisher’s exact test).

Regarding hormonal status, 19 318 women (52.5 %) were cyclic, 14 306 (38.9 %) were menopausal and 3 189 (8.7 %) had missing information. Within the HPVhr-positive subgroup, 54.9 % were cyclic, 36.4 % menopausal and 8.8 % unknown, compared with 52.1 %, 39.2 % and 8.6 %, respectively, among HPVhr-negative women (*P* < 0.001, Fisher’s exact test).

Contraceptive history showed that only 688 women (1.9 %) reported current use of an intra-uterine device (IUD); HPVhr positivity was 1.5 % in IUD users versus 1.9 % in non-users (*P* = 0.094). Leucorrhoea was reported by 1 531 women (4.2 %) with no significant association with HPVhr status (*P* = 0.304). Pre-screening cervical cauterisation (2.0 %) and biopsy (0.8 %) were uncommon, and their frequencies did not differ between HPVhr-positive and -negative groups (cauterisation *P* = 0.103; biopsy *P* = 0.528). Full descriptive statistics are summarised in Table 1.

**Table 1:** Characteristics of the study population according to high-risk HPV (HPVhr) Status (*n* = 36,813)

Characteristic	Total (*n* = 36,813)	HPVhr Negative (*n* = 32,225; 87.5%)	HPVhr Positive (*n* = 4,588; 12.5%)	*P*
**Age (mean ± SD)**	46.14 ± 8.65 years	46.28 ± 8.56 years	45.12 ± 9.21 years	< 0.001*
**Age group, *n* (%)** 25–29 years old 30–65 years old	448 (1.2%) 36,365 (98.8%)	307 (1%) 31,918 (99%)	141 (3.1%) 4,447 (96.9%)	< 0.001**
**Hormonal status, *n* (%)** Cyclic Menopause Unknown	19,318 (52.5%) 14,306 (38.9%) 3,189 (8.7%)	16,801 (52.1%) 12,638 (39.2%) 2,786 (8.6%)	2,517 (54.9%) 1,668 (36.4%) 403 (8.8%)	< 0.001**
**IUD *n* (%)** Absent Present	36,125 (98.1%) 688 (1.9%)	31,608(98.1%) 617 (1.9%)	4,517 (98.5%) 71 (1.5%)	0.094**
**Leucorrhea *n* (%)** Absent Present	35,282 (95.8%) 1531 (4.2%)	30,898 (95.9%) 1327 (4.1%)	4,384 (95.6%) 204 (4.4%)	0.304**
**Genital history** **Pre – screening** **Cervical cauterization *n* (%)** Absent Present	36,085 (98%) 728 (2%)	31,573 (98%) 652 (2%)	4,512 (98.3%) 76 (1.7%)	0.103**
**Cervical biopsies *n* (%)** Absent Present	36,523 (99.2%) 290 (0.8%)	31,967(99.2%) 258 (0.8%)	4,556 (99.3%) 32 (0.7%)	0.528**

Note: SD, standard deviation; *Student T-Test, **Fisher's Exact Test

## DISCUSSION

In this large, region-wide screening cohort, 12.5 % of women were HPVhr-positive, a prevalence consistent with recent European estimates for primary HPV-based programmes. This proportion reflects the underlying burden of HPVhr infection in the South-Muntenia region and highlights the importance of comprehensive, organized screening approaches.

In many developed countries, HPVhr testing has already replaced the Pap test as the primary screening method, regardless of age [14]. The recognition of persistent HPVhr infection as a necessary step in cervical carcinogenesis has reshaped screening strategies. Initially introduced as a triage method for abnormal cytology results, HPV testing subsequently became an adjunct (co-testing) and is now widely adopted as a stand-alone tool for primary screening. The superiority of HPVhr-based screening over cytology alone is well established, offering key advantages:

*Increased sensitivity:* detection of the presence of HPVhr (single or cumulative genotype) identifies women at high risk of high-grade dysplasia and CC, and leads to additional testing and treatments before turning into an invasive cancer.

*Risk stratification performance*: persistent HPVhr infection (clinically defined as two or more positive HPVhr tests within 12 months) is necessary for carcinogenesis and therefore identifies women at increased risk of cervical dysplasia.

*Reduction in unnecessary colposcopies:* Evidence shows that co-testing (HPVhr + Pap) leads to more colposcopies without a corresponding increase in invasive cancer detection, highlighting the benefit of HPVhr-first strategies in minimizing overdiagnosis and overtreatment [15,16].

Our findings therefore make three contributions to the evolving landscape of HPV-based cervical cancer prevention.

First, the 12.5 % HPVhr prevalence observed in >36 000 women from South-Muntenia is fully aligned with recent European population-screening estimates (range 10–14 %), confirming that Romania’s burden is comparable to its regional peers despite historically lower coverage. The disproportionate positivity among 25- to 29-year-olds underscores the need to strengthen catch-up vaccination and provide tailored communication for younger women who enter organized screening later than recommended.

Second, the absence of significant associations with IUD use, leucorrhea or prior cervical procedures supports the current guideline emphasis on virological rather than purely gynecological risk factors when stratifying women for follow-up. The positive correlation with cyclic hormonal status, while modest, warrants additional investigation into the interplay between endogenous estrogen, cervico-vaginal immunity and HPV persistence.

Third, this real-world demonstration that a primary HPVhr algorithm can be implemented across seven counties—with centralized PCR testing, electronic data capture and rapid result feedback—offers a scalable template for other middle-income settings. Leveraging these operational lessons could accelerate Romania’s progress towards the WHO 90-70-90 targets of 90 % vaccination, 70 % twice-lifetime screening and 90 % treatment coverage by 2030.

### Strengths and limitations

The study benefits from its large, region-representative cohort and harmonized laboratory workflow. However, its cross-sectional design precludes incidence or clearance analyses; self-reported variables may be subject to recall bias; and the sample under-represents women <30 years. Longitudinal follow-up—including genotype-specific persistence and cost-effectiveness modelling—has already commenced and will be reported separately.

## CONCLUSION

Early diagnosis, supported by appropriate treatment and longitudinal monitoring, is critical for interrupting the initiation and progression of cervical cancer. The identification of individual risk factors, particularly in the early stages, allows for the timely initiation of personalized interventions.

In this large screening cohort, high-risk human papillomavirus (HPVhr) infection was primarily observed in women aged 30 to 65 years, those with cyclic hormonal status, and those without intrauterine devices, leucorrhea, or prior cervical procedures. These findings reinforce the established role of persistent HPVhr infection as a central risk factor in CC pathogenesis.

The implementation of primary HPVhr screening within the SCCUT program demonstrated a feasible and effective model for early detection. Broader adoption of such programs, alongside increased public awareness and vaccination efforts, is essential for reducing disease burden and supporting global elimination goals. In Romania, overcoming socio-cultural barriers remains a critical priority for ensuring equitable access to prevention and care.
